# Response to Letter to the Editor: Impact of 1024‐Matrix Size on Perforating Artery Visualization in Cerebral CTA Using a 64‐Slice CT Scanner

**DOI:** 10.1002/jmrs.70102

**Published:** 2026-07-11

**Authors:** Hokuto Nagumo, Yuki Nagata, Mayumi Maruko, Jun Sakai, Yusuke Fujiwara, Joma Oikawa

**Affiliations:** ^1^ Department of Radiology Sapporo Shuyukai Hospital Sapporo Japan; ^2^ Department of Neurosurgery Sapporo Shuyukai Hospital Sapporo Japan; ^3^ Department of Radiological Technology Japan Healthcare University Sapporo Japan

## Abstract

This response addresses a Letter to the Editor commenting on our article on 1024‐matrix reconstruction for perforating artery visualisation in cerebral CTA using a 64‐slice CT scanner (https://doi.org/10.1002/jmrs.70055). It acknowledges that integration with iterative or deep‐learning reconstruction, workflow and PACS burden, and downstream clinical outcomes warrant further study, and emphasises that a targeted 1024‐matrix strategy limited to clinically critical intracranial regions offers a practical compromise.

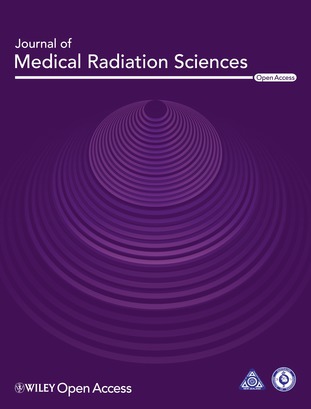

We sincerely thank Liu and Wu for their thoughtful letter regarding our article on the impact of 1024‐matrix reconstruction for perforating artery visualisation in cerebral CTA using a conventional 64‐slice CT scanner [1]. Their careful reading and constructive perspectives are greatly appreciated, and we welcome the opportunity to respond.

We appreciate all three points raised by Liu and Wu, which closely align with the limitations acknowledged in our original manuscript.

Regarding iterative and deep learning‐based reconstruction, we agree that generalisability beyond filtered back projection (FBP) is an essential next step. In our study, we deliberately employed FBP to isolate the effect of matrix size itself, without confounding contributions from noise‐reduction algorithms. However, we recognise that iterative reconstruction is the de facto standard in routine clinical practice, and the noise‐reduction benefits of advanced reconstruction methods should not be overlooked. Indeed, recent studies have demonstrated that combining super‐resolution deep learning reconstruction with a 1024‐matrix on conventional scanners improves image sharpness and diagnostic quality for abdominal CTA [2], and that deep learning reconstruction optimised for brain CTA significantly enhances perforator vessel delineation on ultra‐high‐resolution CT [3]. Investigating how such advanced reconstruction algorithms interact with increased matrix size on conventional 64‐slice platforms from multiple vendors represents an important and promising direction for future research.

Regarding workflow and PACS impact, we acknowledge that our study did not quantify the practical burden of increased data volume. As Liu and Wu correctly note, 1024‐matrix reconstruction quadruples the image data in the axial plane, which may necessitate dedicated post‐processing workstations and attention to network transfer capacity. A targeted 1024‐matrix reconstruction strategy limited to clinically critical intracranial regions, as we suggested in our original manuscript and as Liu and Wu also propose, appears to be a practical and workflow‐friendly compromise. Systematic evaluation of reconstruction time, data transfer speed and post‐processing efficiency in routine clinical workflows is a valuable line of enquiry, and we would welcome contributions from the community in this area.

Regarding clinical outcomes, we agree that our study assessed only image‐based visualisation quality and did not evaluate downstream surgical or neurological outcomes. The clinical importance of this question is well illustrated by previous reports showing that surgical clipping of anterior choroidal artery aneurysms carries a non‐negligible risk of treatment‐related ischaemic complications due to injury to perforating arteries [4]. Whether improved preoperative visualisation of perforating arteries through 1024‐matrix reconstruction can contribute to more accurate surgical planning and reduced vascular injury is a question we consider highly worthwhile. We hope to pursue such evaluations in the future, while recognising that prospective studies with meaningful clinical follow‐up will require substantial multidisciplinary collaboration.

In closing, we thank Liu and Wu once again for their constructive engagement with our work. Their letter reinforces the clinical relevance of matrix size optimisation on widely available 64‐slice CT scanners, and we hope it will stimulate further validation studies across vendors, reconstruction algorithms and clinical endpoints.

## Funding

The authors have nothing to report.

## Ethics Statement

The study was conducted in accordance with the Declaration of Helsinki, and approved by the Ethics Committee of Sapporo Shuyukai Hospital (protocol code 2023–14 and date of approval 12 March 2024).

## Consent

This retrospective study used anonymised data from patients who had provided comprehensive informed consent for the use of their medical information in research studies at Sapporo Shuyukai Hospital. All personal identifiers were removed from the data before analysis to protect patient privacy and confidentiality in accordance with ethical guidelines.

## Conflicts of Interest

The authors declare no conflicts of interest.

## Linked Articles

This article is linked to Nagumo et al. papers. To view this article, visit https://doi.org/10.1002/jmrs.70055.

## Data Availability

The authors have nothing to report.
